# Testicular Toxicity of Water Pipe Smoke Exposure in Mice and the Effect of Treatment with Nootkatone Thereon

**DOI:** 10.1155/2019/2416935

**Published:** 2019-06-25

**Authors:** Badreldin H. Ali, Suhail Al-Salam, Sirin A. Adham, Khalid Al Balushi, Mohammed Al Za'abi, Sumaya Beegam, Priya Yuvaraju, Priyadarsini Manoj, Abderrahim Nemmar

**Affiliations:** ^1^Department of Pharmacology and Clinical Pharmacy, College of Medicine and Health Sciences, Sultan Qaboos University, Muscat, Oman; ^2^Department of Pathology, College of Medicine and Health Sciences, UAE University, Al Ain, UAE; ^3^Department of Biology, College of Science, Sultan Qaboos University, Muscat, Oman; ^4^Department of Physiology, College of Medicine and Health Sciences, UAE University, Al Ain, UAE

## Abstract

There is a worldwide increase in the popularity of water pipe (shisha) tobacco smoking including in Europe and North America. However, little is known about the effects of water pipe smoke (WPS) exposure on male reproductivity. We have recently demonstrated that WPS exposure in mice induces testicular toxicity including inflammation and oxidative stress. Nootkatone, a sesquiterpenoid found in grapefruit, has antioxidant and anti-inflammatory effects. However, the possible protective effect of nootkatone on WPS-induced testicular toxicity has not been reported before. Here, we tested the effects of treatment of mice with nootkatone on WPS-induced testicular toxicity. Mice were exposed to normal air or WPS (30 minutes/day, for 30 days). Nootkatone (90 mg/kg) was given orally to mice by gavage, 1 h before WPS or air exposure. Nootkatone treatment significantly ameliorated the WPS-induced increase in plasma levels of inhibin, uric acid, and lactate dehydrogenase activity. Nootkatone also significantly mitigated the decrease in testosterone, androgen-binding protein, and estradiol concentrations in the plasma induced by WPS. In testicular homogenates, WPS exposure caused a decrease in the total nitric oxide level and an increase in the proinflammatory cytokine interleukin-1*β* level and oxidative stress markers including malondialdehyde, cytochrome C, and 8-Oxo-2′-deoxyguanosine. All the latter effects were significantly alleviated by nootkatone treatment. Moreover, in testicular homogenate, nootkatone inhibited the expression of nuclear factor-kappaB induced by WPS. Likewise, histological examination of mouse testes showed that nootkatone treatment ameliorated the deterioration of spermatogenesis induced by WPS exposure. We conclude that nootkatone ameliorated the WPS-induced testicular inflammation and oxidative stress and hormonal and spermatogenesis alterations.

## 1. Introduction

Tobacco consumption is an established major public health problem that results in substantial morbidity and mortality [1, 2]. Water pipe smoking (WPS) (also termed *huqqa*, *sheesha*, *nargilah*, *hubble-bubble*, and *qalyan*), an ancient and common method of tobacco consumption in Asia and North Africa [3, 4], is regaining widespread global popularity, especially among the young population in Western countries [5, 6]. It involves passage of air that is heated by charcoal across tobacco flavored by or sweetened with either fruit or molasses sugar, which makes the smoke more aromatic than cigarette smoke. Contrary to common belief, the smoke produced from the heated tobacco in WPS is equally or even more toxic than cigarette smoke [7, 8]. WPS has been proven to be more genotoxic than cigarette smoking [9].

Nootkatone (C_15_H_22_O) is a sesquiterpenoid isolated from some plants such as grapefruit and rhizomes of *Cyperus rotundus* and has been reported before to significantly mitigate DNA damage in mice exposed to diesel exhaust particles, and thrombogenicity and systemic and cardiac oxidative stresses, by mechanisms which may include heme oxygenase-1 and nuclear factor erythroid-derived 2-like 2 activation [10]. It has also been shown to possess anti-inflammatory and antioxidant actions in lung tissues of mice [11].

We have previously reported that subacute and chronic WPS exposure exerts deleterious effects on the testes of mice [12, 13]. These adverse effects included reductions in the plasma concentrations of some reproductive hormones and increased oxidative stress and inflammation biomarkers in plasma and testes. As nootkatone is reported to counteract the latter two actions [10, 11], it was of interest to investigate if treatment of WPS-exposed mice with this agent would ameliorate the testicular toxicity of WPS.

## 2. Materials and Methods

### 2.1. Animals and WPS Exposure

This research was reviewed and sanctioned by the Institutional Review Board of the College of Medicine and Health Sciences (UAEU), and experiments were conducted according to the protocols approved by the Institutional Animal Care and Research Advisory Committee.

Thirty-two male BALB/c mice (Taconic Farms Inc., Germantown, NY, USA) were housed in a conventional animal house at controlled temperature (at 26°C) and humidity of 60% and maintained on a 12-hour light-dark cycle (lights on at 6 : 00 am). Mice were randomly placed, eight each, in a plastic cage and were given *ad libitum* supply of water and a standard pellet diet. The animals were left to acclimatize for a week and then were randomly divided into four equal groups: air-exposed (control), WPS-exposed, nootkatone-treated (90 mg/kg, by oral gavage), and exposed to WPS and treated with nootkatone (90 mg/kg, by oral gavage) groups. The treatments were given for 30 min daily for 30 consecutive days. This dose was selected from previous research conducted in our laboratory on nootkatone [10].

Mice were positioned in soft restraints and connected to the exposure tower [13]. The animals were exposed to either WPS or air through their noses using a nose-only exposure system connected to a water pipe (inExpose System, SCIREQ, Canada). The details of the exposure were reported in a previous publication (e.g., [14]). Mice were exposed to a commercial product of apple-flavored tobacco (Al Fakher Tobacco Trading, Ajman, UAE). Tobacco was lit with an instant light charcoal disk (Star, 3.5 cm diameter and 1 cm width). As is the case for human use, the smoke from the water pipe first passes through the water before it is drawn into the exposure tower. The exposure regimen is controlled by a computerized system (inExpose System, SCIREQ, Canada). A computer-controlled puff was generated every minute, leading to a two-second puff duration of WPS exposure followed by 58 s of fresh air. The duration of each exposure session was 30 min/day.

Following the last exposure session to WPS or air, mice in the four groups were immediately placed in metabolic cages and urine of each mouse was collected over a 24 h period and the volume was measured. Immediately after urine collection, mice were anesthetized with sodium pentobarbital (45 mg/kg, i.p.) and blood was collected from the inferior vena cava in tubes with the anticoagulant ethylenediaminetetraacetic acid (EDTA) (4%). The collected blood was spun at 4°C for 15 min at 900 × *g*, and the plasma obtained was stored at −80°C to await analysis.

Mice were then sacrificed with an overdose of anesthesia. The testes from all animals were collected and rinsed with ice-cold PBS (pH 7.4) and weighed. The left and half of the right testis were immediately frozen at -80°C pending biochemical and molecular analyses. The other half of the right testis was used for histologic al studies.

### 2.2. Biochemical Tests in Plasma

Androgen-binding protein (ABP) and inhibin concentrations in plasma were measured by ELISA kits purchased from BioSource company (San Diego, CA, USA) and CUSABIO (Hubei, Wuhan, China), respectively. Plasma uric acid was measured using an autoanalyzer BS-120 (Mindray, Shenzhen, China). Lactate dehydrogenase (LDH) activity was measured using a kit from Abcam (Cambridge, UK).

### 2.3. Urine Cotinine Analysis

The concentration of cotinine, the nicotine metabolite in urine, was measured by an ELISA kit from Creative Diagnostics (Shirley, NY, USA).

### 2.4. Assessment of Testicular Oxidant/Antioxidant Status

The testes were dissected out, and the right testis from each mouse was thoroughly washed with ice-cold normal saline, weighed, and minced. Part of the testis was homogenized (10% *w*/*v*) in cold potassium phosphate buffer (pH 7.4, 0.05 M), and the homogenate was centrifuged at 1500 g for 10 min at 4°C. Thereafter, a colorimetric kit from BioVision (Milpitas, CA, USA) was used for the estimation of lipid peroxidation as malondialdehyde (MDA) concentration in the testicular supernatant. Total nitric oxide (NO) and nitrite/nitrate in the testicular supernatant were measured using a kit from R&D Systems (Minneapolis, MN, USA). Testicular protein was estimated using the BCA Protein Quantitation Kit from BioVision (Milpitas, CA, USA).

Part of the testicular homogenate was centrifuged at 9391 g for 30 min at 4°C. Cytosolic fraction (supernatant) obtained was used to measure cytochrome C using an R&D Systems ELISA kit (Minneapolis, MN, USA). The remaining supernatant was centrifuged at 10000 g for 20 min at 4°C. The supernatant obtained was further centrifuged at 12000 g for 20 min at 4°C to obtain the postmitochondrial supernatant, which was used for the estimation of interleukin-1beta (IL-1*β*) and 8-hydroxy-2′-deoxyguanosine (8-OHdG) using ELISA kits purchased from Thermo Fisher Scientific (Waltham, MA, USA) and BioVision (Milpitas, CA, USA), respectively.

### 2.5. Histopathological Assessment

A piece of the testis was taken from randomly selected five controls and six WPS-exposed animals, five nootkatone-treated animals, and six mice treated with either NK or nootkatone + WPS and placed first in Bouin's fluid for an hour, then transferred to 10% formalin, and processed as described before [13]. Four *μ*m sections were prepared from paraffin blocks and stained with hematoxylin and eosin.

The stained sections were assessed in a blinded fashion by a pathologist using an Olympus microscope (EX41, Tokyo, Japan). Spermatogenesis was evaluated using Johnsen's mean testicular biopsy score (MTBS) criteria [15]. A score of 1–10 was given to each tubule according to germ cell maturation (1, neither germ cells nor Sertoli cells are present; 2, Sertoli cells without germ cells; 3, only spermatogonia; 4, only a few spermatocytes; 5, many spermatocytes; 6, only a few early spermatids; 7, no spermatozoa but many spermatids are present; 8, only a few spermatozoa are present; 9, many spermatozoa are present but spermatogenesis is disorganized; and 10, complete spermatogenesis and perfect tubules). For these evaluations, MTBS was calculated in 100 tubules of each testes using an Olympus E41 Microscope.

### 2.6. Western Blotting Technique

Testis protein expression for NF-*κ*B was estimated using Western blotting technique. Briefly, testis tissues collected from the mice were immediately snap frozen with liquid nitrogen and stored at −80°C. Later, the tissues were weighed, rinsed with saline, and homogenized with lysis buffer (Cell Signaling Technology, USA). Protease and phosphatase inhibitors (Sigma, Berlin, Germany) were added to the protein lysate samples. The homogenates were centrifuged for 20 min at 4°C. Protein estimation was done in the supernatants collected using a Pierce bicinchoninic acid (BCA) protein assay kit (Thermo Fisher, Waltham, MA, USA). Protein lysate samples were all adjusted to have 100 *μ*g of total protein per sample and were electrophoretically separated using 12% sodium dodecyl sulfate polyacrylamide gel electrophoresis (SDS-PAGE) and then transferred onto polyvinylidene difluoride membranes (Bio-Rad, Hercules, CA, USA). The immunoblots were then blocked with 5% nonfat milk and subsequently probed with either the NF-*κ*B p65 (ab ab16502) or the GAPDH (ab8245) (Abcam, UK) at a 1 : 1000 dilution, incubated at 4°C as a normalizing internal control overnight. The blots were then washed three times for 5 minutes and incubated with goat anti-rabbit IgG horseradish peroxidase-conjugated secondary antibody at 1 : 5000 dilutions (Abcam, Cambridge, UK) for 2 h at room temperature and developed using the clarity Western ECL Substrate (Bio-Rad, Hercules, CA, USA). The densitometric analysis of the protein bands was performed using the Image Lab™ software (Bio-Rad, USA).

### 2.7. Immunofluorescence

Mouse testes were embedded in paraffin blocks, and sections 3 mm thick were placed on positively charged slides. The slides were deparaffinized and dehydrated, and antigen retrieval was done in 200 mM EDTA solution at pH 9 for 30 minutes at 90°C. The activity of endogenous peroxidases was blocked by adding 2% hydrogen peroxide for 15 min. The slides were washed twice in phosphate buffer saline (PBS) (Sigma, Germany) and then in PBS + 0.05% Triton X-100 for 5 min each. The slides were incubated with a blocking solution of 5% normal goat serum for 30 min at room temperature and then incubated overnight at 4°C with anti-8-hydroxy-2′-deoxyguanosine antibody (ab48508) diluted in antibody diluent (Dako, USA). After washing 3 times with 1 × PBS (Sigma, Germany), the slides were incubated with (1 : 500) secondary antibody (anti-rabbit conjugated to Alexa Fluor® 488 fluorescent dye) (Cell Signaling, USA) for 1 hour. DAPI (Cell Signaling Technology), the nuclear stain (1250), was added for 3 min. Finally, slides were mounted by fluorescent mounting media (Dako, USA) and visualized under the fluorescent microscope (Nikon H600L), with digital a camera (DS-Ri2) and the imaging software NIS-Elements version 4.40.

### 2.8. Drugs, Chemicals, and Kits

Nootkatone was brought from Sigma-Aldrich (St. Louis, MI, USA). All the other chemicals used were of the highest grade available. The sources of each kit used were mentioned above.

### 2.9. Statistical Analysis

Values obtained are reported as mean ± standard error of the mean (SEM). Statistical significance was assessed by one-way analysis of variance (ANOVA) followed by Bonferroni's multiple comparison tests using GraphPad Prism software, version 5.03. To ascertain if parameters were normally distributed, the KD normality test was applied and *P* < 0.05 was considered significant.

## 3. Results

### 3.1. Body and Testicular Weight

WPS exposure, with or without nootkatone, did not significantly affect the body weight of treated mice or their absolute testis weights. WPS exposure, however, slightly increased the relative testis weight, an effect that was significantly mitigated by nootkatone concomitant treatment.

### 3.2. Urinary Cotinine Concentration

Table 1 depicts the concentration of the nicotine metabolite, cotinine, in urine of controls, WPS-exposed, nootkatone-treated, and WPS-exposed + nootkatone-treated mice. The cotinine urinary level in WPS-exposed mice was 191% higher than that in control mice. Coadministration of WPS and nootkatone reduced the level to 98.5%.

### 3.3. Plasma Analytes

**Figure 1:**
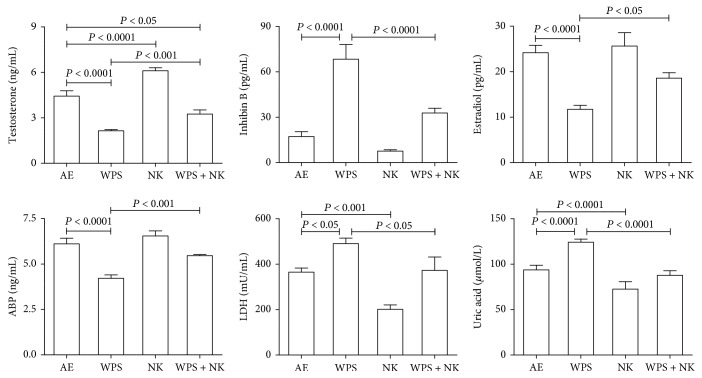
The plasma concentrations of testosterone, inhibin B, estradiol, androgen-binding protein (ABP), lactate dehydrogenase (LDH), and uric acid in mice exposed to normal air (AE) or water pipe smoking (WPS) (30 min/day) with or without treatment with nootkatone (NK) (90 mg/kg/day). Each vertical column with a bar represents the mean ± SEM (from 8 mice in each group). *P* less than 0.05 was considered significant.

As shown in Figure 1, WPS exposure significantly increased the concentrations of inhibin and uric acid, as well as the activity of LDH. The exposure also significantly decreased the plasma concentrations of testosterone, androgen-binding protein, and estradiol (*P* < 0.0001). Nootkatone treatment significantly ameliorated the WPS-induced increase in plasma levels of inhibin, uric acid, and lactate dehydrogenase activity. Nootkatone also significantly mitigated the decrease in testosterone, androgen-binding protein, and estradiol concentrations in the plasma induced by WPS.

### 3.4. Assessment of Testicular Inflammatory and Oxidative Stress Status

**Figure 2:**
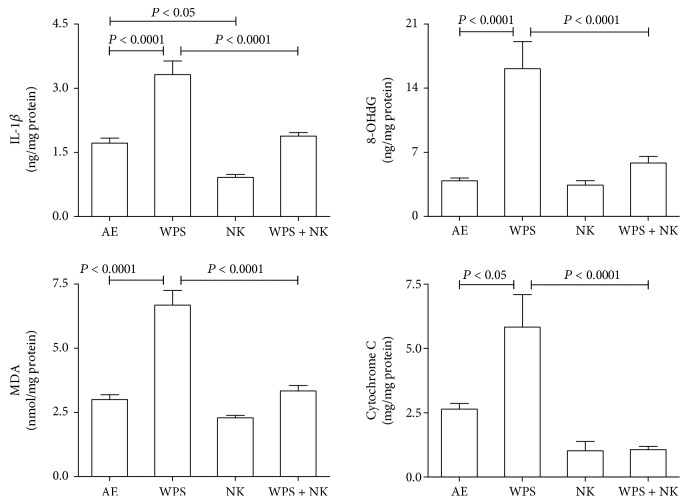
The testicular homogenate concentrations of interleukin-1*β* (IL-1*β*), 8-Oxo-2′-deoxyguanosine (8-OHdG), malondialdehyde (MDA), and cytochrome C in mice exposed to normal air (AE) or water pipe smoking (WPS) (30 min/day) with or without treatment with nootkatone (NK) (90 mg/kg/day). Each vertical column with a bar represents the mean ± SEM (from 8 mice in each group). *P* less than 0.05 was considered significant.

As shown in Figure 2, WPS exposure significantly increased the concentrations of IL-1*β*, 8-OHdG, MDA, and cytochrome C when compared to the controls (*P* < 0.05–*P* < 0.0001). Nootkatone treatment alone significantly reduced the inflammatory cytokine (*P* < 0.05) when compared to the control and did not affect the other testicular analytes measured. Simultaneous exposure to WPS and nootkatone treatment significantly mitigated the increase in the four measured analytes induced by WPS exposure (*P* < 0.05–*P* < 0.0001).

### 3.5. Assessment of Testicular Nitrosative Stress Status

**Figure 3:**
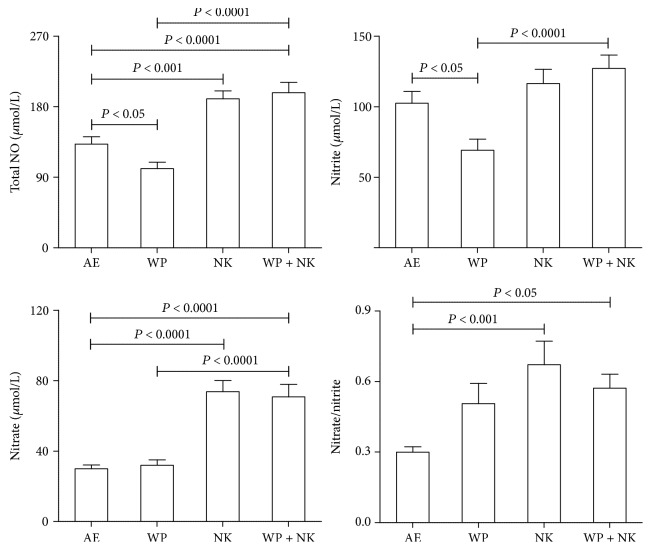
The concentrations of nitrite, nitrate, total nitric oxide, and nitrite/nitrate in testicular homogenates of mice exposed to normal air (AE) or water pipe smoking (WPS) (30 min/day) with or without treatment with nootkatone (NK) (90 mg/kg/day). Each vertical column with a bar represents the mean ± SEM (from 7 mice in each group). *P* less than 0.05 was considered significant.

These results are shown in Figure 3. WPS exposure significantly reduced total NO and nitrite levels (*P* < 0.05–*P* < 0.001) but did not significantly affect nitrate levels, when compared to the controls. WPS exposure raised the ratio of nitrate to nitrite insignificantly. Concomitant exposure to WPS and nootkatone treatment significantly mitigated the decrease in the total NO and nitrite analytes induced by WPS exposure (*P* < 0.05–*P* < 0.0001).

### 3.6. WPS and the Proinflammatory Marker NF-*κ*B p65

**Figure 4:**
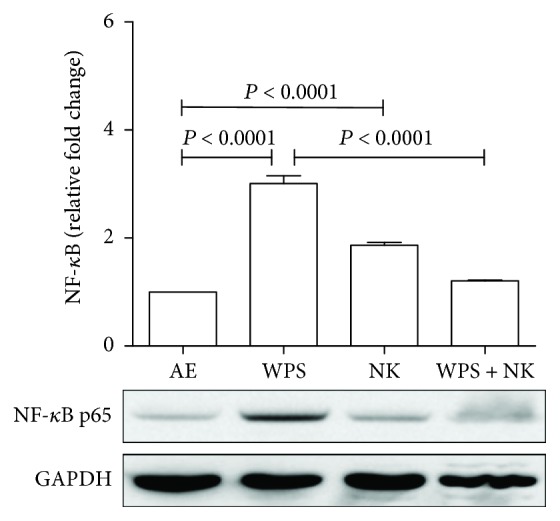
Representative Western blots indicated that proinflammatory NF-*κ*B was increased in the mouse testes as a result of WPS exposure and nootkatone reduced its effect. Densitometry quantification of two independent blots using Image Lab software showed that the NF-*κ*B in the testes of mice exposed to WPS was significantly increased compared to that of the control and/or nootkatone-treated mice. Nootkatone caused a slight increase in NF-*κ*B. However, its use along with WPS exposure caused a significant reduction in mice treated with nootkatone and exposed to WPS. The error bars represent the standard error of the mean of two independent experimental replicates. *P* less than 0.05 was considered significant.

Western blot analysis shown in Figure 4 indicated a significant increase in the proinflammatory marker NF-*κ*B p65 in the testes of mice exposed to WPS. Nootkatone treatment alone showed a slight increase in NF-*κ*B when compared with that from the untreated controls. Concomitant administration of nootkatone to mice exposed to WPS significantly reduced the levels of NF-*κ*B p65 compared to those expressed in the testes exposed to WPS alone (*P* < 0.0001).

### 3.7. DNA Oxidative Damage in Testicular Tissue

**Figure 5:**
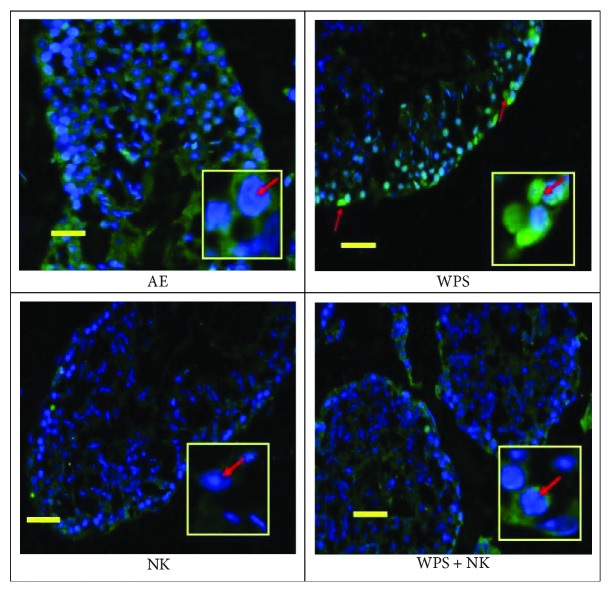
DNA oxidation damage was observed in mouse testes as a result of WPS use in mice. 8-Oxo-2′-deoxyguanosine/DAPI staining for mouse testes showed no nuclear disposition in control mice (a); an increase in the DNA oxidation represented by the increase in the intensity of 8-Oxo-dG (fluorescent green stain) in the nucleus of spermatocytes of the water pipe smoke- (WPS-) treated mice (b). The use of nootkatone in mice exposed to air (c) and the use of nootkatone for the mice exposed to WPS reduced the oxidation damage, and there was no nuclear disposition of 8-Oxo-dG (d).

As shown in Figure 5, the DNA oxidative damage in the immunofluorescent-stained testis sections was measured by quantifying the nuclear staining of 8-Oxo-2′-deoxyguanosine (8-Oxo-dG) represented by the green fluorescence in the nucleus. The latter overlapped with the nuclear stain DAPI in the testes of mice exposed to WPS but was not detected in controls, mice treated with nootkatone alone, or mice treated with nootkatone plus WPS exposure.

Control (AE) and nootkatone-treated mice showed considerably less oxidative damage than that seen in WPS-exposed mice. WPS markedly increased the DNA oxidative damage, represented by the increase in the intensity of 8-Oxo-dG (fluorescent green stain), evident in the nucleus of spermatocytes of WPS-treated mice. WPS-exposed mice treated with nootkatone had substantially less oxidative damage than WPS-exposed mice.

### 3.8. Histopathology

**Figure 6:**
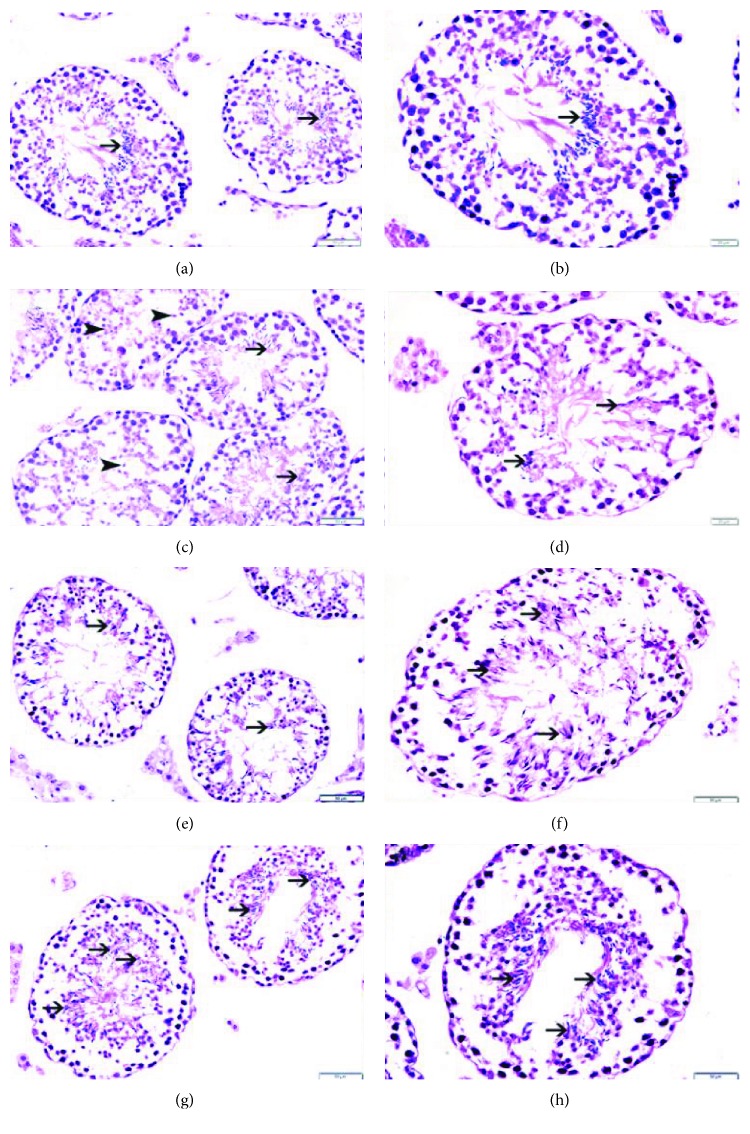
Histopathological examination of mouse testes in the air (control), water pipe smoke (WPS), nootkatone (NK), and WPS + NK groups: (a, b) representative sections from testes of the control group showing well-maintained spermatogenesis with spermatozoa (thin arrow) in seminiferous tubules; (c, d) representative sections from testes of the WPS group showing well-maintained spermatogenesis with spermatozoa (thin arrow) in seminiferous tubules, while some of the tubules show no spermatozoa but spermatids in seminiferous tubules (arrowhead); (e, f) representative sections from testes of the NK group showing well-maintained spermatogenesis with spermatozoa (thin arrow) in seminiferous tubules; (g, h) representative sections from testes of the WP + NK group showing a well-maintained spermatogenesis with spermatozoa (thin arrow) in seminiferous tubules.

Figure 6 illustrates the histopathological examination of mouse testes in the 4 studied groups. In the control group, there was a complete spermatogenesis in 31% of the seminiferous tubules (score 10) (Table 2). In other areas of the control group, many spermatozoa were present but there was disorganized spermatogenesis in 38% of the seminiferous tubules (score 9) (Table 2). Moreover, only a few spermatozoa were present in 19% of the seminiferous tubules (score 8) (Table 2). The spermatogenesis was affected in some of the seminiferous tubules in which 12% of the tubules showed no spermatozoa and where there was a block differentiation at the spermatid level (Table 2). The overall MTBS score of the control group is 8.8.

In the WPS group, there was a complete spermatogenesis in 14% of the seminiferous tubules (score 10) (Table 2). In other areas of the WPS group, many spermatozoa were present but there was disorganized spermatogenesis in 31% of the seminiferous tubules (score 9) (Table 2). Also, only a few spermatozoa were present in 35% of the seminiferous tubules (score 8) (Table 2). The spermatogenesis was affected in some of the seminiferous tubules in which 20% of the tubules showed no spermatozoa and where there was a block differentiation at the spermatid level (Table 2). The overall MTBS score of the WPS group is 8.31.

In the NK group, there was a complete spermatogenesis in 33% of the seminiferous tubules (score 10) (Table 2). In other areas of the NK group, many spermatozoa were present but there was disorganized spermatogenesis in 33% of the seminiferous tubules (score 9) (Table 2). Furthermore, only a few spermatozoa were present in 19% of the seminiferous tubules (score 8) (Table 2). The spermatogenesis was affected in some of the seminiferous tubules in which 15% of the tubules showed no spermatozoa and where there was a block differentiation at the spermatid level (Table 2). The overall MTBS score of the NK group is 8.79.

In the WPS + NK group, there was a complete spermatogenesis in 30% of the seminiferous tubules (score 10) (Table 2). In other areas of the WPS + NK group, many spermatozoa were present but there was disorganized spermatogenesis in 28% of the seminiferous tubules (score 9) (Table 2). Furthermore, only a few spermatozoa were present in 26% of the seminiferous tubules (score 8) (Table 2). The spermatogenesis was affected in some of the seminiferous tubules in which 16% of the tubules showed no spermatozoa and where there was a block differentiation at the spermatid level (Table 2). The overall MTBS score of the WPS + NK group is 8.66.

## 4. Discussion

In this work, 30 min daily exposure of mice for one month to WPS significantly decreased the plasma concentrations of testosterone, estradiol, and androgen-binding protein and increased that of inhibin B and uric acid and LDH activity. These results confirm and extend the previously reported deleterious effects of WPS on male reproduction in mice [12, 13]. As far as we are aware, there are no data on the effect of WPS on human reproduction. The previous research in humans have shown that the information about the effect of cigarette smoking on male and female sex hormones is at variance (reviewed by [16]). In view of the current wide use of WPS in many regions of the world, the effect of active and passive WPS exposure on various aspects of human reproduction is warranted, especially in countries where pregnant women are commonly exposed to passive smoking or use cigarettes and/or WPS [17].

Uric acid was measured in the plasma in this work because it is known to cause endothelial dysfunction, which is an important feature for erectile dysfunction [18]. The latter occurs via decreased NO production. Our results showed that testicular NO concentration is decreased in WPS-exposed mice. This is a direct evidence for the implication of both NO and uric acid in the deleterious effects of WPS on male reproduction in mice. It has been suggested that addiction to cigarette smoking (CS) involves inhaled NO from CS, in addition to endogenous NO released from nervous tissue following stimulation of nicotinic acetylcholine receptors by nicotine, and that CS causes dysfunction in the endothelial nitric oxide synthase [19]. The same possible mechanism may occur with WPS.

Previously we found a significant increase in LDH activity in bronchoalveolar lavage (BAL) fluid of mice exposed to WPS, suggesting cytolysis, and proteins in BAL fluid, reflecting increased epithelial permeability [20]. In the present study, WPS exposure significantly increased the LDH activity in plasma, an action that was ameliorated by nootkatone. Such effect has never been reported before. LDH activity in plasma and testes has long been known to positively correlate with exposure to toxicants. In humans, CS has been shown to increase LDH activity in serum and saliva, suggesting that it can be used as an indicator of tissue damage in the oral cavity [21]. However, no such action has been reported in humans exposed to WPS.

In this work, we found that WPS exposure significantly increases the oxidative and nitrosative free radicals and increased lipid peroxidation, as assessed by malondialdehyde measurement. Oxidative and nitrosative stresses are known to be associated with deleterious effects on male reproduction, as they disrupt the integrity of sperm DNA and diminish the fertilizing potential of the reproductive cells due to collateral damage inflicted upon proteins and lipids in the sperm plasma membrane [22]. Excess free radical load due to increased reactive oxygen species and nitric oxide generation may cause severe testicular oxidative damage by causing peroxidation of lipids and formation of carbonyls [23].

We evaluated DNA damage in the testicular homogenates by measuring 8-OHdG levels. 8-OHdG is one of the prominent forms of free radical-induced oxidative lesions in DNA and has therefore been widely used as a biomarker of nucleic damage owing to oxidative damage [24]. It has also been used as a reliable biomarker for measuring oxidative damage, detecting male infertility, and investigating sperm DNA fragmentation [25].

Cytochrome C plays a principal role in the electron transport chain in mitochondria. It has been shown that exposure of mice to cigarette smoke [26] or WPS [27] induces oxidative stress causing a rise in the expression or release of cytochrome C in cardiac cells, suggesting mitochondrial damage. Here, we have shown that cytochrome C concentration is also elevated in testicular homogenates of mice exposed to WPS, an action that was significantly reversed by nootkatone.

The NF-*κ*B p65 level was also found to be increased by WPS exposure. This protein complex controls transcription of DNA, cytokine production, and cell survival and plays an important role in several pathophysiological processes such as immune reaction, inflammation, and apoptosis [28]. It is possible that an inflammatory response is triggered due to an oxidative stress in the tissues (including testes) in response to WPS (as indicated in this work by the significant elevation of IL-1*β* in plasma). This confirms the role of redox-sensitive transcription factors in the pathway of signaling the proinflammatory mediators such as NF-*κ*B [29]. We have recently shown that WPS increases the expression of NF-*κ*B in lung tissues and exercise training inhibits the signaling pathways that lead to activation of NF-*κ*B [28]. The inhibitory action of nootkatone on signaling pathways that lead to activation of NF-*κ*B has been reported before in isolated HaCaT cells [30].

Our work revealed a significant increase in testicular 8-OHdG after WPS exposure, an action that was significantly reversed by concomitant treatment with nootkatone. It has recently been confirmed that the use of WPS in healthy humans significantly increases this DNA damage marker [31]. Moreover, the histological examination of testes revealed that WPS induces alteration of spermatogenesis and that nootkatone treatment ameliorated this effect. Such finding has never been reported before.

In the present study, we did not measure nicotine concentration in our experimental animals due to several technical challenges. Instead, we measured the urinary cotinine concentrations in all of these mice. Cotinine is the main tobacco-specific metabolite of nicotine and is considered an established marker of tobacco exposure, whereby subjects with higher levels of cotinine are considered to be more exposed to nicotine [32].

We conclude that nootkatone ameliorated the WPS-induced testicular inflammation and oxidative stress and hormonal and spermatogenesis alterations. Our study provides experimental evidence that the use of nootkatone, pending further pharmacological and toxicological studies, can be considered a useful agent and could have the potential to alleviate the testicular toxicity induced by WPS.

## Figures and Tables

**Table 1:** Effect of treatment with nootkatone on the urine cotinine level in mice daily exposed to water pipe smoke for 30 days.

Group	Cotinine (ng/mL)
AE	0.66 ± 0.14
WPS	1.92 ± 0.21^a^
NK	0.78 ± 0.12
WPS + NK	1.31 ± 0.10^a,b,*c*^

The values in the table are mean ± SEM (*n* = 7). Nootkatone (90 mg/kg/day) was given to the mice by oral gavage for 30 days. AE: air exposure; WPS: water pipe smoke; NK: nootkatone. Different superscripts indicate significance as follows (*P* < 0.05 was considered significant): a denotes significance of the control group vs. different groups, b denotes significance of the WPS group vs. the WPS + NK-treated group, and c denotes significance of the NK group vs. the WPS + NK-treated group.

**Table 2:** Frequency of Johnsen's mean testicular biopsy score in experimental groups.

Johnsen's score										
Score	10	9	8	7	6	5	4	3	2	1
AE	31%	38%	19%	9%	2%	1%	0%	0%	0%	0%
WPS	14%	31%	35%	16%	2%	2%	0%	0%	0%	0%
NK	33%	33%	19%	11%	2%	2%	0%	0%	0%	0%
WPS + NK	30%	28%	26%	11%	4%	1%	0%	0%	0%	0%

The data are expressed in % of the seminiferous tubules which achieved a particular Johnsen's score in the air exposure (AE), water pipe smoke (WPS), nootkatone (NK), and WPS + NK groups (*n* = 5-6). Nootkatone (90 mg/kg/day) was given to the mice by oral gavage for 30 days, 1 h before WPS or air exposure.

## Data Availability

The data that support the findings of this study are available from Professors Badreldin H. Ali and Abderrahim Nemmar upon reasonable request.
